# An integrative computational systems biology approach identifies differentially regulated dynamic transcriptome signatures which drive the initiation of human T helper cell differentiation

**DOI:** 10.1186/1471-2164-13-572

**Published:** 2012-10-30

**Authors:** Tarmo Äijö, Sanna M Edelman, Tapio Lönnberg, Antti Larjo, Henna Kallionpää, Soile Tuomela, Emilia Engström, Riitta Lahesmaa, Harri Lähdesmäki

**Affiliations:** 1 Department of Signal Processing, Tampere University of Technology, Tampere, Finland; 2 Turku Centre for Biotechnology, University of Turku and Åbo Akademi University, Turku, Finland; 3 Turku Doctoral Programme of Biomedical Sciences, Turku, Finland; 4 Department of Information and Computer Science, Aalto University, Helsinki, Finland

**Keywords:** Lineage commitment, Non-parametric analysis, Th cell differentiation, Time-course transcriptomics, Transcription factor binding

## Abstract

**Background:**

A proper balance between different T helper (Th) cell subsets is necessary for normal functioning of the adaptive immune system. Revealing key genes and pathways driving the differentiation to distinct Th cell lineages provides important insight into underlying molecular mechanisms and new opportunities for modulating the immune response. Previous computational methods to quantify and visualize kinetic differential expression data of three or more lineages to identify reciprocally regulated genes have relied on clustering approaches and regression methods which have time as a factor, but have lacked methods which explicitly model temporal behavior.

**Results:**

We studied transcriptional dynamics of human umbilical cord blood T helper cells cultured in absence and presence of cytokines promoting Th1 or Th2 differentiation. To identify genes that exhibit distinct lineage commitment dynamics and are specific for initiating differentiation to different Th cell subsets, we developed a novel computational methodology (LIGAP) allowing integrative analysis and visualization of multiple lineages over whole time-course profiles. Applying LIGAP to time-course data from multiple Th cell lineages, we identified and experimentally validated several differentially regulated Th cell subset specific genes as well as reciprocally regulated genes. Combining differentially regulated transcriptional profiles with transcription factor binding site and pathway information, we identified previously known and new putative transcriptional mechanisms involved in Th cell subset differentiation. All differentially regulated genes among the lineages together with an implementation of LIGAP are provided as an open-source resource.

**Conclusions:**

The LIGAP method is widely applicable to quantify differential time-course dynamics of many types of datasets and generalizes to any number of conditions. It summarizes all the time-course measurements together with the associated uncertainty for visualization and manual assessment purposes. Here we identified novel human Th subset specific transcripts as well as regulatory mechanisms important for the initiation of the Th cell subset differentiation.

## Background

T cells are key regulators of the adaptive immune system and have a central role in defense against pathogens and cancer as well as protection from autoimmune diseases. CD4^+^ T lymphocytes can differentiate to functionally distinct effector subtypes, including T helper 1 (Th1), T helper 2 (Th2) and more recently described T helper 17 (Th17) cells [1]. Th1 cells secrete effector cytokine IFN-γ and regulate cell-mediated immunity and play a role in the pathogenesis of autoimmune diseases, such as multiple sclerosis. Th2 cells in turn produce IL-4, IL-5, and IL-13 cytokines, and mediate immunity against extracellular pathogens and allergic reactions. Th17 cells, characterized by the production of a proinflammatory cytokine IL-17, regulate inflammatory responses on the mucosal surfaces. For the overall health in humans and animals, the proper balance between different effector T cell types and T regulatory cells is crucial [2,3]. Aberrant activation of Th1 and Th17, or Th2 cells can trigger inflammatory autoimmune diseases as well as asthma and allergy. Previous studies utilizing genome-wide expression data and computational modeling have aimed at revealing the master regulators and regulatory networks within the differentiating Th1 and Th2 cells [4-9]. However, studies in human have been less extensive than in mouse due to the difficulty in collecting sufficient amount of samples to comprehensively profile T cell differentiation over time. In addition, lack of appropriate computational methods suitable for analyzing large-scale experimental data from multiple lineages over several time points spanning the lineage commitment process has limited the progress on revealing dynamics and molecular mechanisms underlying multiple lineage commitment.

A number of different time-series analysis approaches have been proposed to solve large-scale lineage commitment analysis problems. The general purpose *F*-test [10] can be used to test the difference between time-series data sets, but it does not extend to simultaneous comparison of multiple lineages and fails to take into account the correlation between the measurements at different time points. More recent approaches to analyze time-series data, including regression, differential expression, discriminant and clustering methods, are reviewed by Coffey and Hinde [11]. Methods for differential expression analysis include e.g. spline-based methods, generalized *F*-tests and hierarchical error and empirical Bayes models. Spline-based EDGE method by Storey *et al*. [12] is relevant for our problem because it provides comparisons for multiple conditions (lineage commitment profiles). Although EDGE computes a p-value for differential expression, it does not quantify the differential expression for all lineage comparisons, such as reciprocal genes (i.e., all lineages behave differently). ANOVA-based TANOVA method is based on the approach where different ANOVA structures are defined and the optimal one is found by evaluating the effects and significancies of the factors [13]. Recently, Stegle *et al.*[14] proposed an approach based on Gaussian processes (GP) to determine the time interval when a gene is differentially expressed. The methodology of Stegle *et al.* (2010) was limited to analyzing only two conditions. Moreover, it is often observed at transcriptional level that immediately after a treatment, such as activation of T cells by engagement of T cell receptor and CD28, genes are highly dynamic for some time but activity of gene expression decreases at later time points [15,16]. Thus, an ideal computational method − that does not exist at the moment − should take into account the temporal correlation, handle a non-uniform measurement grid, cope with non-stationary processes, and be able to do a well-defined analysis of multiple conditions.

Here we developed a computational methodology, LIGAP (Lineage commitment using Gaussian processes) which analyzes experimental data from any number of lineage commitment time-course profiles and analyzed genome-wide gene expression profiles of human umbilical cord blood T helper cells (Thp) activated through their CD3 and CD28 receptors and cultured in absence (Th0) or presence of cytokines promoting Th1 or Th2 differentiation. The results give insight into differences of the three lineages in the expression landscape and provide marker genes for lineage commitment identification. Key lineage specific, that is, differentially regulated, genes discovered computationally were validated either experimentally at protein level or based on the published literature. Using a module-based analysis, we identified known and putative regulatory control mechanisms by overlaying highly coherent lineage profile clusters with genome-wide transcription factor (TF) binding predictions and pathway information. Consistent with the previously published results on IL-4/STAT6-mediated control of a large fraction of genes in Th2 program [17], our analysis revealed a comparable up-regulated and down-regulated modules, which are suggested to be controlled by STAT6 and other TFs. Interestingly, we also found that the genes which behave differently between all the lineages studied exhibit a consistent characteristic pattern, i.e., they are up-regulated in Th1 polarizing cells, down-regulated in Th2 polarizing cells, and in activated cells (Th0) the expression levels are between Th1 and Th2 cells. In addition, our analysis revealed a large set of novel genes, which are specific for different T cell subsets in human. All the gene expression data and differentially regulated genes as well as software implementing our computational analysis are made publicly available.

## Results

### Experimental data from primary human CD4+ T cells

We used previously published time-course gene expression measurements of activated primary human T cells (Th0) and cells polarized to differentiate to Th2 lineage [17] as well as previously unpublished data set representing Th1 polarizing cells originating from the same naïve Th precursor cells as the Th0 and Th2 cells. The gene expression of Th1 lineage was measured at time points 0, 12, 24, 48 and 72 hours. The measurements from Th0 and Th2 samples were available at the same time points.

### LIGAP: A computational technique to identify condition specific time-course profiles

**Figure 1:**
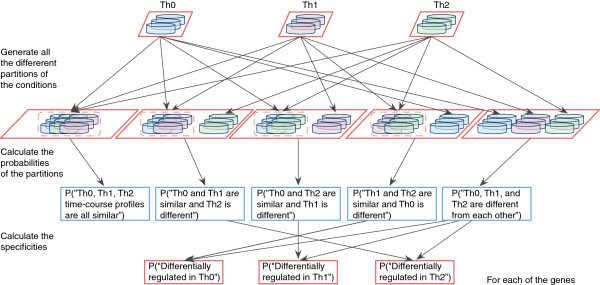
**A schematic illustration of the LIGAP method.** LIGAP implements a statistical analysis of multiple lineage commitment, as shown here, or other time-course profiles. LIGAP considers all possible comparisons between cell subsets, quantifies a probabilistic model fit for each partition, and summarizes the individual probabilities into differential regulation scores. The case of three lineages, Th0, Th1 and Th2 is shown.

The discovery of condition specific genes at the level of gene expression is an important first step in systems biology studies. To capture temporal aspects of biological processes, such as cell differentiation, gene expression is commonly measured over time. We developed a novel model-based method LIGAP for detecting and visualizing changes between multiple lineage commitment time-course profiles. Briefly, for each gene at a time, our method carries out all comparisons between different cell subsets. In the case of Th0, Th1 and Th2 lineages, we assess all 5 alternatives; *(i)* “Th0, Th1, Th2 time-course profiles are all similar” (hypothesis H_1_), *(ii)* “Th0 and Th1 are similar and Th2 is different” (hypothesis H_2_), *(iii)* “Th0 and Th2 are similar and Th1 is different” (hypothesis H_3_), *(iv)* “Th1 and Th2 are similar and Th0 is different” (hypothesis H_4_), and *(v)* “Th0, Th1, and Th2 are all different from each other” (hypothesis H_5_). LIGAP comparisons and quantifications are illustrated in Figure 1. The modeling is done using Gaussian processes, which provide a flexible and nonparametric approach for estimating smooth differentiation profiles. With the help of Bayesian statistics, we can quantify differences and similarities by assigning posterior probabilities for all the different profile comparisons between polarizing cell subsets. The problem can be seen as a model selection problem, where different comparisons are thought of as different model structures (H_1_,… H_5_) and, given experimental lineage commitment profile data D, the marginal likelihood P(D | H_j_), j=1,…,5, is used to score different models. Using the Bayes’ theorem, the marginal likelihoods can be converted into posterior probabilities of different hypothesis. These Bayesian model scores can be used further to quantify genes, which are specific for a certain lineage. For example, the probability of a gene being differentially regulated in Th2 lineage, i.e., score for Th2 is P(“Gene is differentially regulated in Th2” | D) = P(“Th0 and Th1 are similar and Th2 is different” | D) + P(“Th0, Th1 and Th2 are all different” | D) = P(H_2_| D) + P(H_5_| D). Genes which are differentially regulated in each of the conditions can be found by quantifying the probabilities P(“Th0, Th1, and Th2 are all different from each other” | D) = P(H_5_| D) or the three probabilities of differential regulation. Each score quantifies the amount of differential regulation, which refers to distinct temporal behavior from other lineages. The methodology generalizes to any number of lineages/conditions. Our method copes with non-uniform sampling, is able to model non-stationary biological processes (where e.g. changes are fast at the beginning of the experiment and slow at the end), can make comparisons for paired samples, and can carry out the analysis with different number of replicates and missing data. Importantly, the method affords comparison of more than two conditions of interest and is widely applicable to different experimental platforms.

### LIGAP identifies signatures of Th0, Th1 and Th2 cell lineages

**Figure 2:**
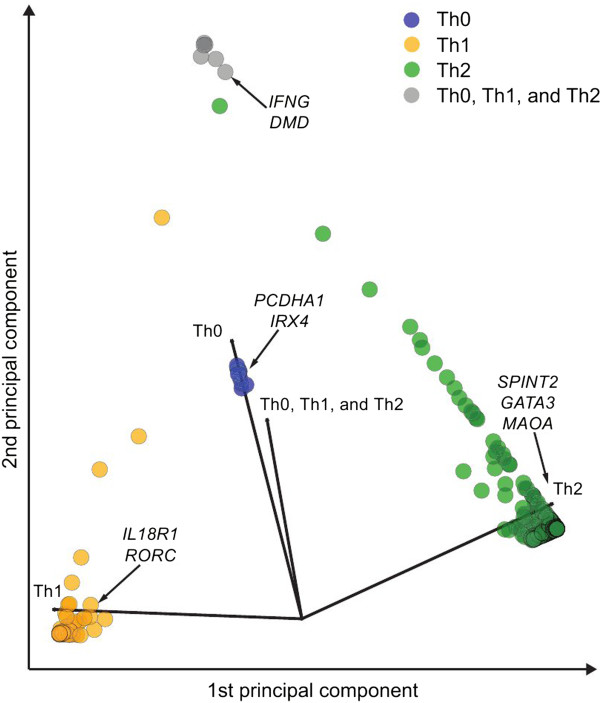
**A two-dimensional PCA visualization of the differentially regulated genes among Th lineages.** Each point corresponds to a differentially regulated gene. The color of the data point indicate the subset specificity as indicated in the figure. Four axes (black arrows) corresponding to different polarizing cell subsets are shown as a reference. The used probability cut-off for each class was 0.9.

We analyzed the genome-wide gene expression time-course data from Th0, Th1 and Th2 lineages using LIGAP. For all genes, the method outputs the posterior probability values for each of the five hypotheses and also computes the scores for genes being differentially regulated in the Th subsets. An overview of the differentially regulated genes is shown in Figure 2, where the four-dimensional data points representing the condition specificities are projected into a plane using the principle component analysis (PCA). This demonstrates the convenience of the presented method as we are able to reduce highly complex data into a meaningful four-dimensional representation using a unified probabilistic framework. In Figure 2 individual points represent different genes and every gene is associated with four probabilities: P(“Differentially regulated in Th0”), P(“Differentially regulated in Th1”), P(“Differentially regulated in Th2”), and P(“Th0, Th1, and Th2 are all different from each other”). Note that IFNγ has the three probabilities P(“Differentially regulated in Th0”), P(“Differentially regulated in Th1”), and P(“Differentially regulated in Th2”) close to unity because the probability P(“Th0, Th1, and Th2 are all different from each other”) is close to unity. We set a criterion (P > 0.9) for the probabilities to call the differentially regulated probe sets; this threshold is in accordance with the Jeffrey’s interpretation of “strong evidence” for the Bayes factor [18]. In addition, we required a minimum of two-fold change between a lineage and all other lineages at some time point during the differentiation for a gene to be called as differentially regulated. The top 49 and 50 gene symbols for Th1 and Th2 lineages, respectively, are listed in Table 1, whereas, the Th0 list includes only 18 genes. In a Additional file 1: Figure S1 are depicted two additional examples illustrating the advantage of considering temporal correlation in gene expression and thus improving the sensitivity of detecting consistent yet subtle changes. In addition, we repeated the analysis using EDGE [12] and TANOVA [13] methods using the default parameter values. TANOVA identified almost twice as many genes (~1,300) to be differentially regulated as LIGAP or TANOVA (~700). A comparison of the obtained ranked lists revealed a higher correspondence between the lists produced by LIGAP and EDGE than with the list produced by TANOVA (data not shown).

**Table 1:** Differentially expressed genes in T cells polarized towards the Th0, Th1 and Th2 subsets

**Top 18 Th0 specific genes**			**Top 49 Th1 specific genes**			**Top 50 Th2 specific genes**		
**Affymetrix probe ID**	**Gene symbol**	**P(“Th0 specific”)**	**Affymetrix probe ID**	**Gene symbol**	**P(“Th1 specific”)**	**Affymetrix probe ID**	**Gene symbol**	**P(“Th2 specific”)**
203881_s_at	DMD(<2)	0.99967984	206618_at	IL18R1(+)	0.999997088	204388_s_at	MAOA(+)	1
223435_s_at	PCDHA1(<2)	0.999832654	203881_s_at	DMD(+)	0.999993101	227006_at	PPP1R14A(+)	1
205027_s_at	MAP3K8(<2)	0.996290375	210354_at	IFNG(+)	0.999987382	205419_at	GPR183(+)	1
228055_at	NAPSB(<2)	0.996196899	207072_at	IL18RAP(+)	0.9999811558	215172_at	PTPN20A(+)	1
220225_at	IRX4(<2)	0.991324709	213943_at	TWIST1(+)	0.999952924	205769_AT	SLC27A2(-)	1
205794_s_at	NOVA1(<2)	0.984972756	222547_at	MAP4K4(<2)	0.999933552	218976_at	DNAJC12(-)	1
221035_s_at	TEX14(<2)	0.981445323	242809_at	IL1RL1(<2)	0.999876515	208510_s_at	PPARG(+)	1
210354_at	IFNG	0.977204551	206007_at	PRG4(<2)	0.999828413	228708_at	RAB27B(+)	1
212012_s_at	KPNA6(<2)	0.976438534	228055_at	NAPSB(+)	0.99973265	45288_at	ABHD6(+)	1
219265_at	MOBKL2B(<2)	0.974857762	235458_at	HAVCR2(+)	0.99959007	229764_at	TPGR1(+)	1
212992_at	AHNAK2(<2)	0.971558242	225955_at	METRIL(+)	0.99954195	235199_at	RNF125(+)	1
223727_at	KCNIP2(<2)	0.968952518	206974_at	CXCR6(+)	0.999495469	203153_at	IFIT1(-)	1
200907_s_at	PALLD(<2)	0.967031439	206785_s_at	KLRC2(<2)	0.99882105	203097_s_at	RAPGEF2(-)	1
1570169_at	CSMD2(<2)	0.950549266	200648_s_at	GLUL(+)	0.997745987	208891_at	DUSP6(+)	1
200906_s_at	PALLD(<2)	0.940149574	205027_s_at	MAP3K8(+)	0.996774094	223159_s_at	NEK6(+)	1
216341_s_at	GNRHR(<2)	0.933169413	225142_at	JHDM1D(<2)	0.996653927	210715_s_at	SPINT2(+)	1
222890_at	CCDC113(<2)	0.921542966	215672_s_at	AHCYL2(<2)	0.996237492	208158_s_at	OSBPL1A(+)	1
201283_s_at	TRAK1(<2)	0.914512473	219383_at	PRR5L(<2)	0.995895316	225752_at	NIPA1(+)	1
			220603_s_at	MCTP2(+)	0.993459782	206638_at	HTR2B(+)	1
			239533_at	GPR155(<2)	0.992909201	205579_at	HRH1(+)	1
			229603_at	BBS12(+)	0.989517632	226508_s_at	TNFSF13B(-)	0.999999999
			237559_at	GPR55(<2)	0.988653639	244413_at	CLECL1(+)	0.999999999
			204284_at	PPP1R3C(<2)	0.988109742	203708_at	PDE4B(-)	0.999999999
			202625_at	LYN(<2)	0.986582272	227438_at	ALPK1(-)	0.999999998
			223767_at	GPR84(<2)	0.984948052	210762_s_at	DLC1(+)	0.999999998
			209348_s_at	MAF(+)	0.983885642	235570_at	RBMS3(+)	0.999999997
			210448_s_at	P2RX5(+)	0.9830704	212077_at	CALD1(+)	0.999999997
			228057_at	DDIT4L(<2)	0.978509028	235301_at	KIAA1324L(+)	0.999999997
			203129_s_at	KIF5C(<2)	0.978078282	209576_at	GNAI1(+)	0.999999997
			1554190_s_at	C10orf81(<2)	0.976976156	1554878_a_at	ABCD3(+)	0.999999996
			228806_at	RORC(+)	0.976176574	205569_at	LAMP3(+)	0.999999996
			227568_at	HECTD2(<2)	0.975089149	205900_at	KRT1(+)	0.999999996
			219753_at	STAG3(+)	0.972731568	210145_at	PLA2G4A(+)	0.999999996
			223374_s_at	B3GALNT1(<2)	0.971777376	227529_s_at	AKAP12(+)	0.999999995
			205098_at	CCR1(<2)	0.969847411	209602_s_at	GATA3(+)	0.999999995
			200907_s_at	PALLD(+)	0.967031845	225566_at	NRP2(+)	0.999999995
			211122_s_at	CXCL11(<2)	0.965307014	1552807_a_at	SIGLEC10(+)	0.999999995
			227697_at	SOCS3(+)	0.965003456	220307_at	CD244(<2)	0.999999994
			212683_at	SLC23A44(<2)	0.960505919	239151_at	BMS1P1(+)	0.999999994
			213572_s_at	SERPINB1(<2)	0.953874496	221241_s_at	BCL214(-)	0.999999993
			244321_at	PGAP1(+)	0.953326576	229625_at	GBP5(-)	0.999999992
			217127_at	CTH(+)	0.947970476	204912_at	IL10RA(+)	0.999999999
			225962_at	ZNRF1(+)	0.944840091	214974_x_at	CXCL5(-)	0.999999989
			219532_at	ELOVL4(<2)	0.937454137	221971_x_at	CTGLF10P(+)	0.999999989
			222838_at	SLAMF7(+)	0.934337899	203853_s_at	GAB2(+)	0.999999989
			228658	MIAT(+)	0.934243107	222457_s_at	LIMA1(+)	0.999999986
			206948_s_at	PMCH(<2)	0.928324396	213385_at	CHN2(+)	0.999999983
			232030_at	KIAA1632(<2)	0.914780073	205630_at	CRH(+)	0.999999979
			213596_at	CASP4(<2)	0.907680384	207861_at	CCL22(+)	0.999999979
						219209_at	IFIH1(-)	0.999999977

Probe sets that fulfill the two-fold change criterion are marked based on the direction of the expression (+ denotes up-regulation and - denotes down-regulation) in the given condition. For example, *IFNγ* expression is enhanced in Th1 compared to Th0 and Th2, whereas expression of *SLC27A2* is decreased in Th2 compared to Th0 and Th1. In addition, probe sets that do not fulfill the fold change criterion are marked in the lists with “<2”. All genes from Th0 and Th1 conditions as well as the top 50 of the Th2 specific genes are shown.

**Figure 3:**
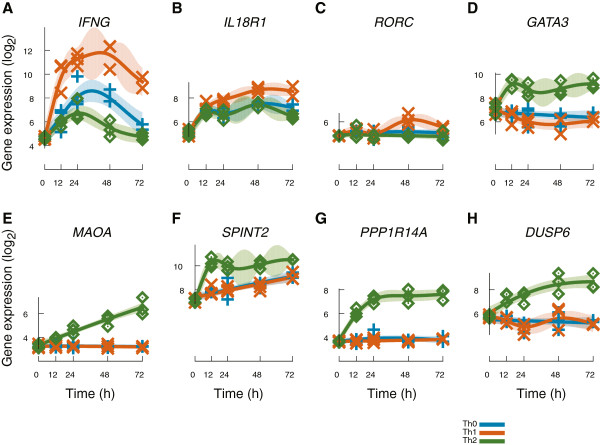
**A detailed visualization of six differentially regulated genes.** X-axis corresponds to time and y-axis shows the normalized log_2_-transformed gene expression values. Solid line denotes the estimated mean expression profile and the shaded area shows the 95% confidence interval. Measurements are shown with individual points. Different lineages are color-coded as shown in the figure. (**A**) *IFNG*, (**B**) *IL18R1*, (**C**) *RORC*, (**D**) *GATA3*, (**E**) *MAOA*, (**F**) *SPINT2*, (**G**) *PPP1R14A*, and (**H**) *DUSP6.*

Our results of the Th subset specific genes agree well with known transcriptional changes during the human T cell differentiation. IFNγ, a hallmark molecule of Th1 lineage, was found to be one of the most significantly up-regulated Th1 specific transcripts (Table 1, Figure 3A, and Additional file 2: Table S1). Furthermore, *IL18R1* encoding the interleukin 18 receptor (IL18R), as well as IL-18 receptor accessory protein (*IL18RAP*) were among the top Th1 specific genes (Table 1, Figure 3B). Expression of IL18R is up-regulated specifically on Th1 cells but not on Th2 cells, thus, IL18R can be regarded as a differentiation marker for Th1 cells [15,19]. In fact, IL-12 and IL-18 can reciprocally up-regulate expression of each other’s receptors in Th1 cells [15,20] and the IL-18 - IL18R system has a significant role in the synergistic effect of IL-12 and IL-18 in triggering efficient NF-κB signaling and enhancement of IFNγ production from human Th1 cells [21]. Intriguingly, in the absence of IL-12, IL-18 has also potential to induce Th2 differentiation and cytokine response [19,22]. The basic helix-loop-helix transcription repressor TWIST1 is also known to be expressed in Th1 cells in IL-12/STAT4, NF-κB and NFAT dependent way and its role has been proposed to be linked to autoregulation of inflammatory cytokine production e.g. IFNγ [23]. Several studies have shown that CXCR6 is predominantly expressed in Th1 cells [24,25] and, inversely, in Th2 prone allergic conditions the expression of CXCR6 was reduced in allergic patients when compared to healthy individuals [26]. Also, an important Th1 linked function has been observed with MAP3K8 as it acts as an upstream activator of ERK via IL-12 and TCR-dependent signaling, promotes expression of T-bet and STAT4, and is actually a STAT4 target itself forming a feedback loop in the Th1 cells [27]. Deficiency in MAP3K8 leads to decreased IFNγ production in T cells and *in vivo* impaired host defense against *Toxoplasma gondii*[27].

Interestingly, the retinoic acid-related orphan receptor gamma (*RORC)* gene encoding RORγt, the key transcription factor in the differentiation program of Th17 cells, was also identified as a Th1 specific gene by the LIGAP analysis (Table 1) as its expression was up-regulated at 48 h time point (Figure 3C). In human, small numbers of T cells producing both IL-17 and IFNγ have been characterized in peripheral blood, in lamina propria of patients with Crohn’s disease as well as in patients with psoriasis [28-30], but currently is it not known how such cells are derived from the naïve precursor cells. Other novel Th1 specific hits identified by the LIGAP include two cytoskeleton associated protein-coding genes dystrophin (*DMD*) and palladin (*PALLD*). *DMD* encodes actin-binding cytoskeletal structure molecule, which has been mostly studied in patients with Duchenne’s muscular dystrophy [31]. These patients develop dystrophin specific autoreactive T cells [31], however, the biological role for dystrophin or palladin in differentiating Th cells is not known. Other genes novel in this context and putatively important for Th1 cell differentiation and/or function include *METRNL*, (meteorin, glial cell differentiation regulator-like), associated with rare cases of Mild ring 17 syndrome [32], *GLUL* encoding a glutamine synthetase, and associated with neuronal disorders and atherosclerotic carotid plaques [33,34], *MCTP2* (multiple C2 domains, trans membrane 2), *BBS12* (Bardet-Biedl syndrome 12), *STAG3* (stromal antigen 3), a meiotic gene, as well as *PGAP1* (post-GPI attachment to proteins 1). *NAPSB* coding for aspartic protease Napsin B is known to be expressed in human spleen and peripheral blood leucocytes, however, it is estimated to be only a transcribed pseudogene [35,36]. Similarly, *MIAT* (myocardial infarction associated transcript) is a non-protein coding gene [37], and the relevance of these transcripts in T cell differentiation is not understood, yet.

**Figure 4:**
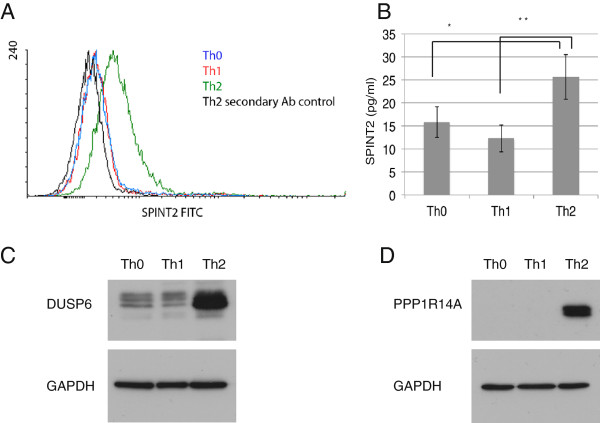
**Experimental validation of characteristic expression of SPINT2, DUSP6 and PPP1R14A in primary human T helper 2 cells.** (**A**) A histogram overlay showing SPINT2 expression at protein level on the cell surface of the Th cells measured with flow cytometer at 24 hours after activation. (**B**) SPINT2 secretion from different T helper cells measured with ELISA at 48 hours after activation. * P<0.05, ** P<0.01. A western-blot images showing (**C**) DUSP6 and (**D**) PPP1R14A expression on Th cells at 72 h post activation. Representatives of three biological replicates are presented. A house keeping protein GAPDH is shown as a loading control.

The top LIGAP hits of Th2 specific genes included several genes with very high probability values (Table 1, and Additional file 2: Table S1) and include a vast number of genes that are both specifically up-regulated and down-regulated in Th2 conditions compared to other Th subsets. Therefore, the list of Th2 specific genes with highest probability is consistent with the previously published results obtained with other computational methods [17]. Importantly, GATA3, the well characterized master transcription regulator of Th2 polarization [38] was identified among the top Th2 hits (Table 1). The transcriptional expression profile of GATA3 was observed to be highly up-regulated at all time points among the cells cultured in Th2 polarizing conditions, whereas the expression profiles in Th0 and Th1 cells exhibited down-regulation (Figure 3D). In addition to well-known subset signature molecules, the analysis identified also a number of poorly characterized molecules in relation to their function in polarized Th cells. Among the highly expressed top 50 Th2 hits, the specificity of these transcripts relative to Th0, but not to Th1, has already been identified at different time points with the standard LIMMA methods (Smyth, [2005]) in the past [17]. One of these Th2 specific top hits was *MAOA*, a gene encoding monoamine oxidase A, whose expression was increasingly up-regulated during the time course (Table 1, Figure 3E). This enzyme degrades amine neurotransmitters, (e.g. dopamine, norepinephrine, and serotonin) and was previously found to be up-regulated in human peripheral blood monocytes after IL-4 and IL-13 stimulation [39] as well as in Th2 cells derived from cord blood naïve CD4+ T cells and, importantly, being indirectly controlled by STAT6 [15,17]. It has been hypothesized that MAOA may act as an anti-inflammatory mediator by degrading serotonin which inhibits generation of TNFα from macrophages and up-regulates phagocytosis [40]. The biological significance of MAOA in Th2 cells, however, remains to be elucidated. Another interesting Th2 specific top hit was *SPINT2* (Table 1, Figure 3F) encoding a transmembrane serine peptidase inhibitor Kunitz type 2 (also called HAI-2 and placental bikunin). SPINT2 was originally named after its homology to hepatocyte growth factor activator inhibitor 1 and its first isolation from human placenta [41,42]. The Kunitz inhibitory domains display potent inhibitory activity towards several trypsin-like serine proteases [43] and mutations in the human *SPINT2* gene cause a broad spectrum of abnormalities in organogenesis [44]. In addition, SPINT2 may function as a tumor suppressor gene, as its mRNA levels are down-regulated in several human cancers (e.g. gliomas, colorectal cancers and liver cancer) and a deficiency in SPINT2 expression is linked with poor prognosis of breast cancer [45]. There are no previous studies where the possible functional role of SPINT2 in human lymphocytes is unraveled, however, *SPINT2* was recently found to be a STAT6 target in human macrophages as well as in human Th2 cells [17,46]. We, hence, chose to experimentally validate the specificity of SPINT2 in primary human Th2-polarizing cells. We tested the specificity of SPINT2 expression at protein level on the cell surface of the Th cells with flow cytometry. At 24 hours after activation and induction of polarization the Th2 cells were found to express significantly more SPINT2 than the Th1 polarizing cells or the activated Th0 cells (Figure 4A). As some of the human SPINT2 transcripts do not harbor the coding signal for the transmembrane domain [47], we therefore also investigated if SPINT2 would be secreted from the Th subsets. The SPINT2 concentrations were measured from the culture supernatants by enzyme-linked immunosorbent assay (ELISA) at 48 hours after activation and polarization, and the Th2 cells were observed to secrete significantly more SPINT2 than Th0 or Th1 cells (Figure 4B). The Th2 specific hits included also *PPP1R14A*, a phosphorylation-dependent inhibitor of smooth muscle myosin phosphatase, involved in regulation of smooth muscle contraction [48] as well as *DUSP6* (dual specificity phosphatase 6), responsible for dephosphorylation of ERK1/2 [49]. Recently, IL-4 induced RNA expression of signaling molecules PPP1R14A and DUSP6 have been reported [15,17,50]. As the regulation of phosphorylation of the signaling intermediates is known to be highly important in defining the cell differentiation, we wanted to experimentally validate the subset specific expression of these two signaling molecules at protein level. We detected a clear Th2 specific PPP1R14A and DUSP6 protein expression at 72 hours time point post activation and initiation of the polarization, and very little or no expression in Th0 and Th1 lineages (Figures 4C and 4D).

### Reciprocal regulators of lineage commitment

In context of determination of T cell subset identity, a key group of genes is the one where the expression kinetics differ between all the lineages. The list of these significantly different genes is shown in Table 2. An illustrative example gene from this list is the well-known Th1 signature cytokine gene *IFNG* (Figure 3A) as well as *TBX21* encoding T-bet, a hallmark transcription factor in Th1 differentiated cells, both of which are also known to suppress Th2 activity [51]. In addition, MAP3K8, FAS, IL12RB2, and IL-26, have been identified to play role in Th1 polarized cells (cf. Table 2). Moreover, Table 2 and Additional file 2: Table S1 contain numerous differentially regulated transcripts which are only poorly characterized or their role in CD4^+^ Th cells has not been studied. The novel Th1 specific genes *DMD* and *PALLD*, encoding cytoskeletal associated proteins dystrophin and palladin, fall into the reciprocally regulated genes in the Th subsets studied here. Also, Th1 specific putative pseudogene *NAPSB* and non-coding transcript *MIAT* show reciprocal transcript profiles. Other novel genes include *PRR5L*, which has been identified to interact with a highly conserved protein kinase TOR (target of rapamycin), a central controller of cell growth and apoptosis [52]. *OSBPL10* encodes oxysterol binding protein-like 10, an intracellular lipid receptor that regulates cellular lipid metabolism [53]. P2RY14 (purinergic receptor P2Y, G-protein coupled, 14) is a membrane receptor for UDP-glucose and plays a role in immune responses in human airway as well as female reproductive track epithelial cells by stimulating cytokine and chemokine production and recruitment of neutrophils [54-56]. P2RY14 has also been identified to function in mouse splenic T cells as a regulator of IL-2 induced proliferation, however, no specific link to Th1 cells has been observed [57]. Also, the significance of *ATP9A* (ATPase, class II, type 9A), *LPAR3* (lysophosphatidic acid receptor 3) functioning in G-protein coupled receptor signaling, *XRN1* (5'-3' exoribonuclease 1), *BSPRY* (B-box and SPRY domain containing), *MCTP2* (multiple C2 domains, transmembrane 2) or *PTPRO* (protein tyrosine phosphatase, receptor type, O) in Th1 cells is yet to be studied. Recent data indicate that in B cells, PTPRO dephosphorylates Syk, a kinase that is critical in signal transduction of B-cell receptor [58].

**Table 2:** The genes whose expression time-courses differ between all the lineages

***Affymetrix probe ID***	***Gene symbol***	Functional annotation*	***Known characteristics in CD4+ T helper cells***
203881_s_at	*DMD(+)*	other	NR
205027_s_at	*MAP3K8(+)*	kinase	required for proper IFNg production [27]
228055_at	*NAPSB(+)*	unknown	NR
210354_at	*IFNG(+)*	cytokine	positive regulation on T cell proliferation, tyrosine phosphorylation of STAT1 and production of IL-12 [59]
200907_s_at	*PALLD(+)*	other	NR
222838_at	*SLAMF7(+)*	other	expression upregulated in Th1 cells[16]
237322_at	*MIAT(+)*	other	NR
1555486_a_at	*PRR5L(+)*	other	NR
209369_at	*ANXA3(+)*	enzyme	expression upregulated in Th1 cells [15]
230109_at	*PDE7B(-)*	enzyme	expression upregulated in Th2 cells [17]
205933_at	*SETBP1(-)*	other	expression upregulated in Th2 cells [17]
216252_x_at	*FAS(+)*	transmembrane receptor	expression regulated by IFNg, important for T cell activation [60,61]
219073_s_at	*OSBPL10(+)*	other	NR
221271_at	*IL21(<2)*	cytokine	important for Th17 and follicular Th cell differentiation, increases activation of STAT3 [62-64]
223475_at	*CRISPLD1(+)*	other	expression upregulated in Th1 cells [16]
206999_at	*IL12RB2(+)*	transmembrane receptor	expression regulated by IFNg, plays a central role in Th1 differentiation [15,65]
200878_at	*EPAS1(+)*	transcription regulator	expression regulated by STAT6 in Th2 cells [17]
206974_at	*CXCR6(+)*	G-protein coupled receptor	predominantly expressed in Th1 cells [24,66,67]
206637_at	*P2RY14(+)*	G-protein coupled 1receptor	NR
212062_at	*ATP9A(+)*	transporter	NR
210029_at	*IDO1(+)*	enzyme	NR
236519_at	*C9orf135(-)*	other	expression upregulated in Th2 cells [17]
214038_at	*CCL8(+)*	cytokine	selectively expressed on Th2 cells, important for trafficking of Th2 cells, required for allergic immune response by Th2 cells [68]
229764_at	*TPRG1(-)*	other	expression upregulated in Th2 cells [17]
231192_at	*LPAR3(+)*	G-protein coupled receptor	NR
202421_at	*IGSF3(-)*	other	expression upregulated in Th2 cells [17]
221111_at	*IL26(<2)*	cytokine	expressed on human Th1 cells, increases activation if human STAT1 and STAT3 [69,70]
227006_at	*PPP1R14A(-)*	other	expression upregulated in Th2 cells [17]
1555785_a_at	*XRN1(+)*	enzyme	NR
230110_at	*MCOLN2(+)*	ion channel	expression upregulated in Th1 cells [16]
222746_s_at	*BSPRY(+)*	other	NR
220684_at	*TBX21(+)*	transcription regulator	expression up-regulated by IL-12, regulates the production of Th1 hallmark cytokine IFNg [71]
220603_s_at	*MCTP2(+)*	other	NR
210839_s_at	*ENPP2(+)*	enzyme	used in homing to secondary lymphoid organs [72]
210164_at	*GZMB(+)*	peptidase	important for TCR-induced cell death in Th2 but not Th1 cells [73]
206126_at	*CXCR5(+)*	G-protein coupled receptor	Il-4 increases expression of CXCR5, important for homing and sensitivity of T cells [74,75]
208121_s_at	*PTPRO(+)*	phosphatase	NR

Probe sets that fulfill the two-fold change criterion are marked with the + and - signs following the gene symbols based on the direction of the expression where + denotes up-regulation and - denotes down-regulation of Th1 specific genes. In addition, probe sets that do not fulfill the fold change criterion are marked in the lists with “<2”. The known associations of genes in Th cell functions or subset specific gene expressions are listed in the table. *^)^ Annotation based on Ingenuity Pathway Analysis ® by Ingenuity Systems. NR, not reported.

The Th2 up-regulated genes, *PDE7B, SETBP1, C9orf135, TPRG1, IGSF3,* or *PPP1R14A* have not been linked to CD4+ Th cell function, although their IL-4 mediated up-regulation has been published, and furthermore, *SETBP1, TPRG1* and *PPP1R14A* have been identified as direct targets of STAT6 [17]. Interestingly, we observed that most of the genes whose expression differs between all the three lineages behave in a similar manner, i.e., they are up-regulated in Th1 and down-regulated in Th2.

**Figure 5:**
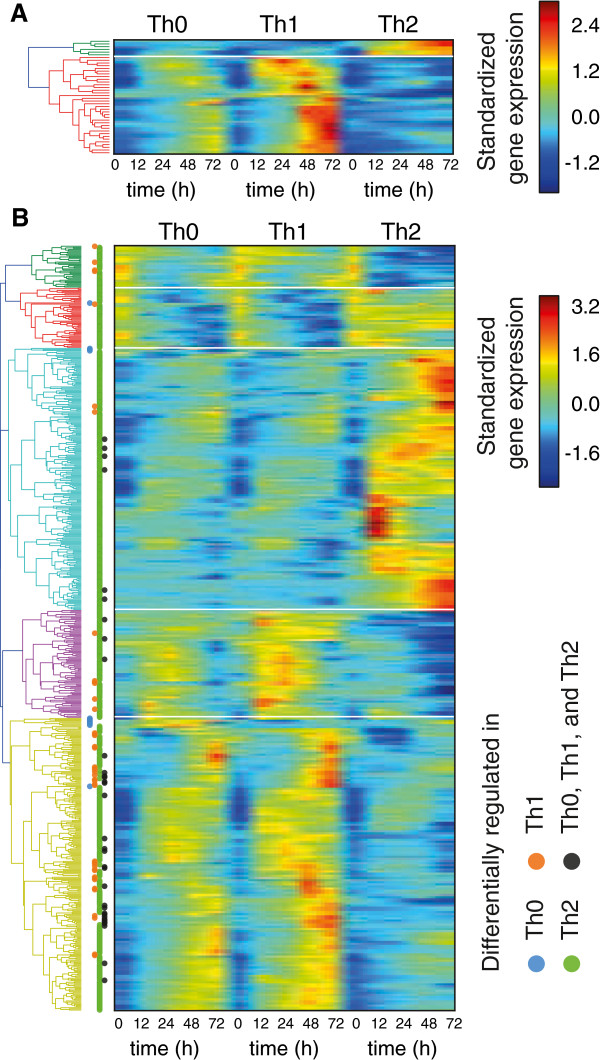
**A global-view of the time-course profiles of reverse regulators of T helper cell differentiation and the time-course profiles of differentially regulated genes.** (**A**) The set of genes that are specific in Th0, Th1, and Th2 are clustered in two clusters. The lower cluster holds altogether 34 unfiltered genes and the upper cluster contains only six genes. Most of the Th0, Th1 and Th2 specific genes are preferentially expressed in Th1 cells and have a lower expression level in Th2 cells. (**B**) The kinetics of the genes LIGAP identified to be differentially regulated are clustered in five clusters. Information about differential regulation is shown with colored dots. Consistent with (**A**), majority of the 34 genes specific in Th0, Th1, and Th2 are assigned to the fifth cluster, whereas the six genes are assigned to the third cluster. (**A and B**) Clusters are indicated with different colors on the branches in the dendrogram and with horizontal white lines in the heat maps. Standardized expression values are shown. The probe set data was standardized across the time points and lineages.

Among the reciprocally regulated genes we found 34 genes up-regulated in Th1 condition and only six genes behaved in the opposite manner. The hierarchical clustering of the kinetic profiles is depicted in Figure 5A. This suggests that there are common mechanisms that induce reverse regulatory behavior. For example, the genes up-regulated in Th1 condition might be controlled downstream of IFNγ. This hypothesis is supported by the clear similarity between the profiles of IFNγ and the profiles of the clustered genes. We prepared a similar figure showing the differences in the kinetics of all the LIGAP identified genes. These results are depicted in Figure 5B and they show the similarity between the Th0 and Th1 lineages and their dissimilarity between the Th2 lineage.

### Transcription factor binding sites in Th2 lineage

To extend our transcriptional analysis into transcriptional regulation, we decided to systematically analyze both genome-wide transcription factor (TF) binding site predictions made *in silico* and comprehensive literature-derived information about target genes of selected TFs. First, we predicted which of the transcription factors have binding sites in the RefSeq gene promoters (defined as [−1000,500] bp around TSS) using the ProbTF tool [76] combined with an empirical p-value computation. We focused on genes that were identified by the previous LIGAP analysis and considered all transcription factors that had known binding specificities (position specific frequency matrices, PSFMs) in TRANSFAC [77] (version 2009.3). We did not restrict our analysis only to those TFs whose transcripts are differentially expressed because, e.g., STAT6 is not differentially expressed during the early differentiation although it is a master regulator in the early differentiation of Th2 cells [17].

An important goal is to identify master regulators of the lineage commitment processes. Recently, it was found out that most of the direct targets of STAT6, an important regulator of Th2 differentiation, were up-regulated in Th2 cells [17]. Here we were interested in identifying TFs whose binding sites are enriched in the promoter regions of the genes which are differentially regulated in Th2 conditions, both among the up-regulated and down-regulated genes. Instead of looking at individual TF binding predictions that are prone to contain false positives, we used the Fisher’s exact test to search for enrichment of binding sites, in comparison to randomly selected gene set. The same analysis was carried out separately for all the differentially regulated gene sets and by taking into account the direction of regulation (repressed or activated).

**Figure 6:**
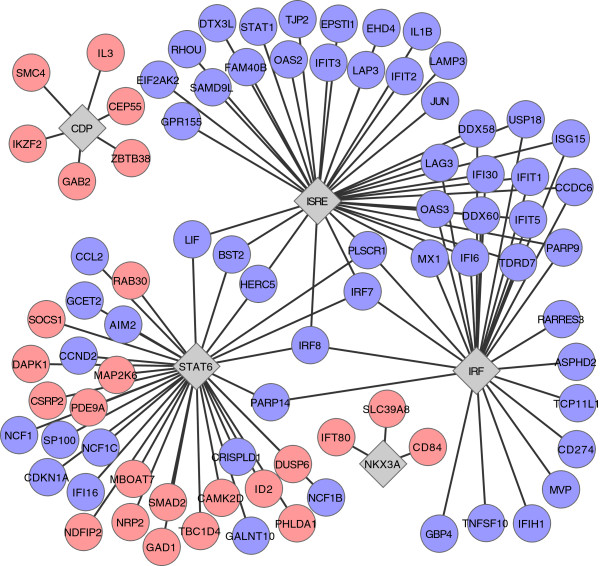
**The network spanned by the enriched transcription factors and their predicted targets.** Altogether five different TF binding family motifs were enriched either among the genes that were either down- or upregulated in Th2 cells. The TFs are depicted using gray tilted squares and the predicted target genes that are up- or down-regulated in Th2 cells are depicted using red or blue circles, respectively.

Using a p-value cut-off of 0.01 for TF binding, we identified three hits from the enrichment analysis among Th2 specific up-regulated genes and three among the Th2 specific down-regulated genes. The results are depicted in Figure 6. The different enriched IRF family motifs were combined and their targets were pooled. In accordance to our previously published results [17], the strongest hit within the Th2 up-regulated genes was STAT6 (p-value = 6.4 e-4), followed by NKX3A (p-value = 4.4 e-3), and CDP (p-value = 9.7 e-3). *NKX3A* is a member of the NKX family of homeobox genes that is expressed in prostate epithelium and functions as a potential prostate tumor suppressor [78]. Recently, in a study focusing on Jurkat cells, a GATA3 binding site on the promoter of *NKX3* gene was identified [79]. Furthermore, in mouse increased expression of *Nkx3a* was observed to be regulated by IL-4 independently of STAT6 [80]. CDP (CCAAT displacement protein) is highly conserved homeodomain transcription factor involved in many cellular processes, including differentiation, development and proliferation [81,82]. Interestingly, CDP has been identified as a repressive regulator of *CD8α* silencer region and *TCRβ* enhancer region and plays a role in promoting repressive chromatin modifications via association with histone deacetylase 1 and histone 3 methyltransferase [83-86]. It is important to notice that the binding motif analysis does not give information about the possible direction of regulation, e.g., it is an open question whether CDP might up-regulate Th2 specific genes or down-regulate the genes in Th0 and Th1 lineages.

The three TF hits having enriched predicted binding sites among the Th2 down-regulated genes were the interferon regulatory factor (IRF) family of TFs (p-value = 2.5 e-6), IFN-stimulated genes factor 3 (ISGF3) (p-value = 1.8 e-4) and STAT6 (p-value = 3.5 e-3). IRF family consists of IRF1 to IRF10 and has been shown to be essential in expression of type I interferon genes, IFN-stimulated genes (ISG) and other pro-inflammatory response related cytokines [87]. These genes are maintained down-regulated during Th2 proliferation and therefore, the results are in line with the Th2 effector cells characteristics [88]. Moreover, IFNγ-induced expression of IRF1 and IRF2 has been shown to directly down-regulate IL-4 production by repressing *IL-4* promoter sites [89]. Opposing to other IRF family proteins, IRF4 has been shown to directly activate IL-4 promoter and IL-10 regulatory elements and be essential in Th2 cell differentiation by influencing the expression of GFI1, a transcriptional repressor in Th2 cells [90-92]. However, the analysis relying on known TF binding specificities will not allow segregation of individual members of the IRF family. Further, an essential regulator of most ISGs is ISGF3 that is composed of STAT1, STAT2 and IRF9 complex and works in conjunction with IRFs [93]. Identification of STAT6 as a regulator among the Th2 down-regulated genes is well in line with our previously published results, although its effect was observed to be less profound within Th2 down-regulated genes than among Th2 up-regulated target genes [17]. Comparison analysis of the predicted STAT6 target genes and Th2 up-regulated and down-regulated genes gave 16 and 19 overlapping genes, respectively. The full lists of overlapping genes are in Additional file 3: Table S2. We further analyzed the correlation between predicted STAT6 target promoters and experimentally observed promoter associated binding sites (Elo *et al*., [2010]), and observed significant correlation (p<0.05) between the target sites. The full list of predicted STAT6 target genes and promoter associated STAT6 binding sites identified by ChIP-seq as well as the overlapping genes are listed in the Additional file 3: Table S2. The overlapping binding sites included promoters for *C14orf177, CISH, HMMR, INO80, MGAT1, NUDCD2, SOCS1, SPINT2* and *ZNF570* genes.

## Discussion

Identification of the key T helper cell regulators provides possible targets for modulation of immune response. To reveal T cell subset specific genes and their often subtle differences in expression, we developed a novel computational method, LIGAP. Traditional ways of identifying differentially expressed genes, such as the *t*-test, are problematic in studying time-series data since there is a need to carry out hypothesis tests on individual time points. On the other hand, commonly used statistical tests for whole time-course, including e.g. F-test, do not account for the inherent correlation between measurement time points. LIGAP overcomes many problems that have previously prevented quantitative comparisons of multiple differentiation profiles, with or without replicates. Among several beneficial features, LIGAP models correlation between time points and can cope with non-stationarities and non-uniform measurement grid. Other methods, such as EDGE, uses splines to estimate smooth time-course profiles but does not quantify the differential expression for all lineage comparisons. TANOVA uses standard regression framework and lacks explicit correlation structure between time points. Our study highlights the validity of the method by identifying known and novel differentially regulated genes and their kinetic differences during T helper cell differentiation. In addition, the non-parametric computational analysis automatically provides informative illustrations of time-course profiles together with associated uncertainty.

LIGAP calculated Th0 specific gene set contains only 18 genes and Th1 specific 49 genes compared to 466 genes that are specific to Th2 conditions. Activation of Thp cells through TCR and CD28 results in induction of IFNγ, which in turn leads to activation of Th1 signature genes. Addition of IL-12, however, results in enhanced induction of these genes and Th1 programming. Consistent with our previous results genes differentially regulated in response to Th1 programming are much more limited than those detected in response to initiation of Th2 response [16,94].

Most of the Th1 specific genes encode well-known Th1 signature molecules. However, also genes new in this context were discovered. Interestingly, we identified *RORC* as one of the Th1 specific genes. Up-regulation of *RORC* in Th1 cells and existence of Th17/Th1 cells, however, remain conflicting as the master regulator of Th1 differentiation, T-bet, is known to inhibit transcription of *RORC* through RUNX1 [95], and expression of IL12Rβ2 is down-regulated by IL-17 [96]. It has been suggested that the high concentration of TGFβ required for *in vitro* Th17 polarization would inhibit IFNγ production [97], hence, it remains an open question whether some conditions would drive the differentiation of IL-17 and IFNγ producing cells from same naïve precursor T cell. Notably, *ex vivo* Th17 cells could be induced to develop further into Th17/Th1 cells by the combined actions of IFNγ and IL-12, and such conditions resulted in permissive chromatin remodeling at the *IL12RB2* locus and loss of repressive histone modification at the *TBX21* locus [29,98].

As an example of previously uncharacterized differentially regulated genes, we validated the expression of Th2-associated phosphatases DUSP6 and PPP1R14A on protein level. PPP1R14A was shown in human pancreatic and melanoma tumor cell lines to positively regulate Ras/MAPK signaling [99], which are also involved in IL-4 induced signaling cascades. In T cells, the ERKs are activated though TCR stimulation and a TCR-mediated activation of Ras/MAPK signaling is required in differentiating murine Th2 but not in Th1 cells [100]. Furthermore, the Ras/MAPK cascade was shown to enhance the stability of GATA3 protein [101] as well as STAT6 independent CD3 and CD28 induced initial IL4 production [102]. DUSP6 on other hand is known to negatively regulate members of the mitogen-activated protein (MAP) kinase superfamily associated with cellular proliferation and differentiation [103]. More specifically, DUSP6 expression was shown to be induced by ERK1/2 signaling in differentiating mouse embryonic cell line and in human retinal pigment epithelial cells [104,105] and it was hypothesized that DUSP6 is an essential part of a negative feedback loop of ERK1/2 signaling [106]. However, the T cell associated functions of both PPP1R14A and DUSP6 are completely unknown. Therefore, their significance in the signaling cascades of differentiating Th2 cells remains a highly interesting area of future research.

*SPINT2* was recently identified as a direct STAT6 target in differentiating human Th2 cells [17] and in this study we are the first to show that SPINT2 is upregulated in Th2 cells at protein level as compared to other Th cell subsets. We found SPINT2 to be specifically expressed on Th2 cell surface as well as secreted into the culture medium, suggesting presence of a multiple transcripts of which some may lack the anchoring transmembrane domain [47]. Human SPINT2 (HAI-2) is a physiological inhibitor of matrix cleaving proteases and decreased expression of SPINT2 has been linked to progression of several cancers [107-109]. Up-regulated expression of extracellular proteases is crucial for pro-cancerous pathways as this enables efficient remodeling of the extracellular matrix as well as cleavage and activation of growth factors and their receptors. Interestingly, a truncated and secreted SPINT2 may act as an inhibitor for the activator of hepatocyte growth factor (HGF) and HGF is prominently expressed in lung tissue and is linked to reduced expression of Th2 cytokines and TGFβ, reduction of allergic airway inflammation, airway hyperresponsiveness and remodeling as well as reduced recruitment of eosinophils to the site of allergic inflammation *in vivo*[110,111]. This suggests that SPINT2 might enhance Th2 response in allergic airway inflammation by inhibiting HGF signaling.

The LIGAP method elegantly identified the reciprocally regulated genes within the Th0, Th1 and Th2 conditions. Essentially, the list included genes encoding the hallmark Th1 specific transcription factor T-bet and cytokine IFNγ as well as the transmembrane receptor for IL-12. This list also included few cytoskeleton associated proteins, such as dystrophin (DMD), and palladin (PALLD), of which there is no current knowledge for their function in differentiating T helper cells. The observation suggests differences in cellular structures or putatively in the interaction of APC with the Th cell subsets as rearrangement of the cytoskeleton in T cells plays an important role in the organization of the immunological synapse (IS) and Th1 and Th2 cells are known to form morphologically distinct ISs [112,113]. In addition to MAP3K8, molecules that participate in phosphorylation signaling cascades e.g. P2RY14, LPAR3, PPP1R14A, and PTPRO suggest their potential role for initiation or regulation of differentiation cascades. Importantly, the results presented here enable opportunities for further data mining and follow-up studies addressing the functions and importance of the novel Th subset specific genes.

The identification of STAT6 as the most significant TF regulating Th2 specific enhancement of transcription by the TF binding analysis is well in line with our previous STAT6 ChIP results [17]. Furthermore, the analysis between the predicted STAT6 target gene promoters and experimentally observed promoter associated binding sites showed statistically significant correlation. Interestingly, the overlapping STAT6 targets included *INO80*, which has been identifies as a part of a chromatin remodeling complex [114] and may hence, be involved in Th2 specific epigenetical regulation of Th cell differentiation. STAT6 specific regulation of Mannosyl (alpha-1,3-)-glycoprotein beta-1,2-N-acetylglucosaminyltransferase (MGAT1), a *N*-glycan-processing enzyme [115], may on one hand be involved in modifying the Th2 cell specific surface glycoprotein structures [116]. The overlapping target sites included also the promoter for *SPINT2*. The number of predicted STAT6 binding sites, however, was much larger than the experimentally observed binding sites, which may reflect the typically observed high false positive rate of computational binding predictions and the cell type specific state of chromatin as well as other competing factors affecting binding *in vitro*. The data created here also further suggests novel control mechanisms involving GATA3 regulated NKX3A as well as chromatin modification associated CDP. Only less than 10% of the Th2 down-regulated genes were reported to be direct targets of STAT6 by Elo *et al*., ([2010]) suggesting other major regulatory mechanisms play role among the IL-4 induced down-regulated genes. We found enrichment of IRF family and ISGF3 binding motifs in promoter regions of genes that are repressed in Th2 polarizing conditions, indicating that these TFs may play a significant role in the suppressing undesired gene expression in differentiating Th2 cells. Indeed, several IRF family members have been identified as differentially expressed during Th cell differentiation and necessary for both Th1 and Th2 polarization. As the IRF family proteins, excluding IRF1, share the same binding specificity model in TRANSFAC, the individual regulatory role for these factors is, however, difficult to postulate based on *in silico* TF binding site analysis.

## Conclusions

The proposed LIGAP method can quantify a well-defined probabilistic specificity score for each gene and for each condition promoting a certain lineage commitment. In addition to grouping and ranking genes based on their dynamics, LIGAP summarizes all time-course measurements, together with the associated uncertainty, in an illustrative summary plot for visualization and manual assessment purposes. While here we have demonstrated the utility of LIGAP in analysis of gene expression dynamics, the LIGAP method is widely applicable to many types of datasets including quantitative time-course experiments and generalizes to any number of conditions.

## Methods

***Human CD4+ T cell purification and culturing.*** The human naïve umbilical cord blood CD4+ T cells were isolated as previously described [17]. Briefly, umbilical cord blood was collected from healthy neonates born in Turku University Hospital, Finland. Mononuclear cells were separated with Ficoll-Paque gradient centrifugation (#GEHE17-1440-3, Amersham Biosciences) and CD4+ T cells were then isolated with magnetic beads (Dynal CD4 Positive Isolation Kit, #113-31D, Invitrogen). After isolation the CD4+ cells were pooled to prepare cell cultures consisting cells from several neonates. The same pooled cells as utilized for Th0 (activated) and Th2 (activated and IL-4 stimulated) culture conditions by Elo *et al*. ([2010]) were used parallel for Th1 polarizing cultures. For activation, the cells were treated with plate-bound anti-CD3 (500 ng/24-well culture plate well, #IM1304, Immunotech) and soluble anti-CD28 (500 ng/ml, #IM1376, Immunotech) in density of 2-4 × 10^6^ cells/ml of Yssel’s medium (Iscove modified Dulbecco medium, #31980-048, Invitrogen) supplemented with Yssel medium concentrate [117], 1% human AB serum (#C11-011, PAA) and 100 U/ml Penicillin and 100 μg/ml Streptomycin (#P0781, Sigma) at 37°C in 5% CO2. For induction of Th1 cell polarization, IL-12 (2.5 ng/ml, # 219-IL, R&D Systems) was added to the cultures. At 48h after activation, IL-2 was added (17 ng/ml, #202-IL, R&D Systems) to all the cells and the polarizing conditions were maintained throughout the culture. The polarizing Th cells were harvested at time points 0, 12, 24, 48 hours in three replicates and at 72 hours in two replicates.

All the data included in this manuscript has been acquired under the permission from the Ethics Committee of the Hospital District of Southwest Finland approving the anonymous collection of cord blood samples after a parental consent, and the permission being in compliance with the Helsinki Declaration

***Microarray studies.*** The preparation of samples for microarray detections was done as described in [17]. Essentially, total RNA (RNeasy Mini Kit, Qiagen) was extracted from the cultured cells and cRNA hybridized on Affymetrix GeneChip HG-U133 Plus 2.0 arrays (Affymetrix, Santa Clara, USA). All the microarray samples included in this study have been prepared at Finnish DNA Microarray Centre, Turku. The raw microarray data were processed using robust multi-array average normalization and log2-transformed in R (version 2.12.0) using the Bioconductor affy package (version 1.28.0).

***Flow cytometry***. The Th0, Th1 and Th2 condition cells at 24 hours were stained for SPINT2 expression studies. Purified anti-SPINT2 (8.7 μg/ml, #HPA 011101-100UL, Sigma-Aldrich) was used as primary antibody followed by staining with FITC-conjugated F(ab’)2 anti-rabbit IgG secondary antibody (1:1000 dilution, #11-4839-81, eBioscience). The stainings were analyzed with LSR II flow cytometer (BD Biosciences) and Flowing Software (http://www.flowingsoftware.com).

***ELISA.*** The cell culture supernatants (at 48 hours) from Th0, Th1 and Th2 conditions were assayed for SPINT2/HAI-2 secretion by ELISA (# DY1106, R&D) according to the manufacturer instructions.

***LIGAP.*** We construct our model-based lineage commitment comparison and visualization methodology, called LIGAP, using non-parametric GP regression similar to that in [14], extend the methodology to any number of conditions and propose to use a non-stationary neural network (NN) covariance function k(x^p^,x^q^) = σ*asin(x^p^ '*diag(l^-2^)*x^q^ / sqrt[(1+x^p^'*diag(l^-2^)*x^p^)*(1+x^q^'*diag(l^-2^)*x^q^)]). The vectors x^p^ and x^q^ are augmented by an extra bias unit value entry and the parameter l defines the length-scale and σ controls the signal variance [118]. A non-stationary covariance function is chosen because often after cell activation or other stimulation the effects on temporal behavior of gene expression are very active and dynamic right after the stimulation but they mellow down over time and, thus, the observed behavior is non-stationary. For each gene at a time, LIGAP makes all comparisons between different cell subsets over the whole time-course data sets. In our application, the multiple hypotheses H_j_ are defined by the different partitions of the cell lineages. For example, if there are only two different lineages, then there are two different partitions (or hypothesis): H_1_ denotes that lineages are similar and H_2_ denotes that lineages are different. In our application consisting of three lineages, Th0, Th1 and Th2, we have 5 alternative hypotheses; *(i)* “Th0, Th1, Th2 time-course profiles are all similar” (hypothesis H_1_), *(ii)* “Th0 and Th1 are similar and Th2 is different” (hypothesis H_2_), *(iii)* “Th0 and Th2 are similar and Th1 is different” (hypothesis H_3_), *(iv)* “Th1 and Th2 are similar and Th0 is different” (hypothesis H_4_), and *(v)* “Th0, Th1, and Th2 are different from each other” (hypothesis H_5_). LIGAP comparisons and quantifications are illustrated in Figure 1. In general, the total number of different partitions of N lineages is known in literature as the Bell number B_n_ (e.g., B_1_ = 1, B_2_ =2, B_3_ = 5, B_4_ = 15, etc.) [119].

Bayes factor is commonly used to see the evidence of the two alternative hypotheses; differentially expressed or not within a given time interval. To extend this to multiple lineages, we use the marginal likelihood p(D_i_ | H_j_) to define the posterior probabilities of the different hypotheses H_j_. For each of the hypothesis H_j_, the data D_i_ for the i^th^ gene is split according to the partitioning. For example, for our application containing three lineages, hypothesis H_1_ corresponds to grouping data from all lineages, hypothesis H_2_ corresponds to splitting the data so that Th0 and Th1 time-course profiles are grouped together and time-course profiles from Th2 forms its own subset of data, hypothesis H_3_ corresponds to splitting the data so that Th0 and Th2 time-course profiles are grouped together and Th1 forms its own subset of data, etc.

For each hypothesis, non-parametric regression is carried out separately for each subset of the data. For example, for the hypothesis H_3_ we fit a GP to the combination of Th0 and Th2 time-course profiles and another GP to the Th1 time-course profiles. Following the standard GP regression methodology [118], the marginalization is done over the latent regression function and the hyperparameters are estimated using type II maximum likelihood estimation with a conjugate gradient based optimization algorithm initiated with ten randomly chosen hyperparameter values. Under the assumption of Gaussian likelihood and noise, the marginal likelihood can be written out analytically, and thus its value can be easily evaluated [118]. The marginal likelihood of a certain hypothesis (i.e., partitioning) is the product of the marginal likelihood of the separate subsets. The key idea behind the modeling is to find the marginal likelihood of the data under different hypotheses and thus have a probabilistic score to objectively compare different hypotheses.

Using the Bayes’ theorem and assuming unbiased, equal prior probabilities for different hypotheses (i.e., P(H_k_) = P(H_l_) for all k and l), we can write the posterior probabilities for the i^th^ gene as P(H_j_ | D_i_) = P(D_i_ | H_j_)P(H_j_)/C, where C = Σ_j_ P(D_i_ | H_j_)P(H_j_) is a normalizing constant. Finally, these quantities can be combined to quantify the score of differential regulation for each gene. For example, the probability of the i^th^ gene being differentially regulated in Th2 lineage can be quantified as P(“Gene is differentially regulated in Th2” | D_i_) = P(H_2_ | D_i_) + P(H_5_ | D_i_) .

***ProbTF*.** ProbTF [76] method is used to make TF binding predictions on promoters of all RefSeq genes. Sequence specificities of TFs are taken from the TRANSFAC database [77] version 2009.3. All non-redundant PSFMs associated to human were taken, totaling 248 matrices. Promoters are defined as the [−1000,500] bp region around TSS. To assess statistical significance, we construct a TF specific null distribution by randomly sampling 50000 genomic locations of size 1501 nucleotides, against which the p-values of TF binding are computed.

***Hierarchical clustering.*** The hierarchical clustering in Figure 5 was done using complete linkage and Euclidean distance metric.

***Data access.*** The data discussed in this publication have been deposited in NCBI's Gene Expression Omnibus [120] and are accessible through GEO Series accession number GSE 32959 (http://www.ncbi.nlm.nih.gov/geo/query/acc.cgi?acc=GSE32959).

## Competing interests

The authors report no financial or non-financial competing interests relevant to the subject of this article.

## Authors' contributions

TÄ, and HL designed the LIGAP method. TÄ implemented the LIGAP method, and normalized, integrated, and analyzed the gene expression data. AL provided the transcription factor binding predictions. SME, TL, HK, and ST did the data mining and experimental validations. EE optimized the ELISA detections and participated in validations. SME, TÄ, TL, HK, RL, and HL drafted and finalized the manuscript. All authors read and approved the final manuscript. RL, and HL led, designed and supervised the study.

## Supplementary Material

Additional file 1 **Figure S1.** Additional data file 1 is a PDF containing two panels illustrating the sensitivity of Sorad to identify changes in time series by integrating modest changes between time series over time. Click here for file

Additional file 2 **Table S1.** The following additional data are available with the online version of this paper. Additional data file 2 is a table showing the full results of the LIGAP analysis. Click here for file

Additional file 3 **Table S2.** Additional data file 3 is a table listing the enriched transcription factor binding motifs in the set of Th2 specific genes and the overlapping genes, and the full listing of predicted STAT6 binding sites and the overlapping genes with the published promoter associated STAT6 targets. Click here for file
