# Supplementary figures and images for: An integrative computational systems biology approach identifies differentially regulated dynamic transcriptome signatures which drive the initiation of human T helper cell differentiation
Source: BMC Genomics. 2012 Oct 30;13:572. doi: 10.1186/1471-2164-13-572 (PMC3526425; doi:10.1186/1471-2164-13-572)

**A**

GZMB / 210164\_at

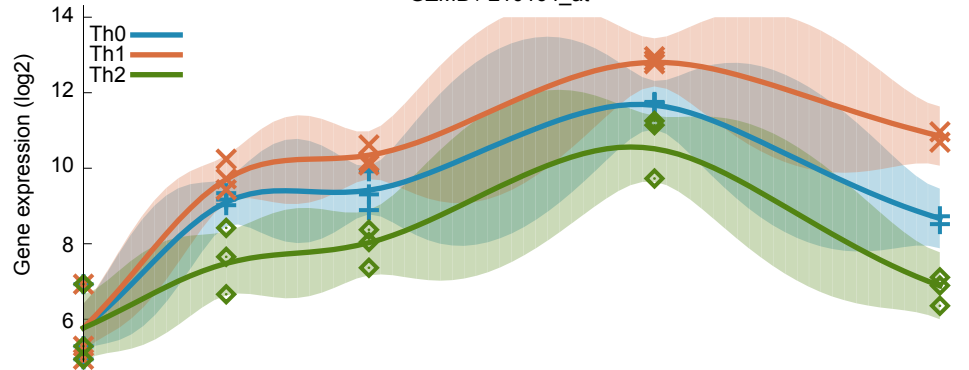**B**

SLC25A44 / 212683\_at

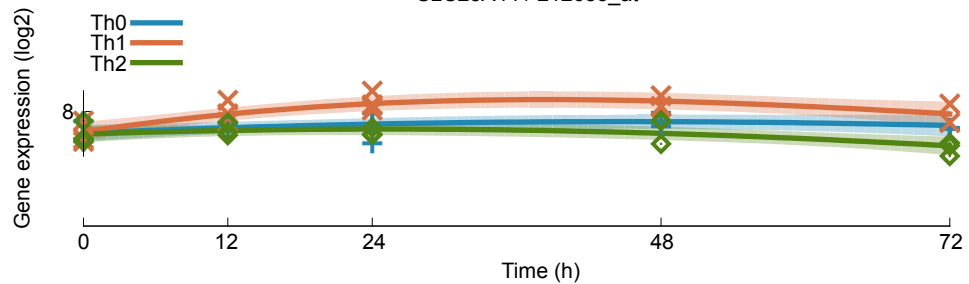

Supplement: Additional file 1 — Figure S1. Additional data file 1 is a PDF containing two panels illustrating the sensitivity of Sorad to identify changes in time series by integrating modest changes between time series over time. [file 1471-2164-13-572-S1.pdf]
